# Efficacy of Chinese Herbal Injections for the Treatment of Primary Nephrotic Syndrome: A Bayesian Network Meta-Analysis of Randomized Controlled Trials

**DOI:** 10.3389/fphar.2020.579241

**Published:** 2020-10-16

**Authors:** Hangxing Yu, Miaoru Han, Wei Lin, Lin Wang, Panying Liu, Kang Yang, Ming Pei, Hongtao Yang

**Affiliations:** Department of Nephrology, The First Teaching Hospital of Tianjin University of Traditional Chinese Medicine, Tianjin, China

**Keywords:** network meta-analysis, Bayesian model, Chinese herbal injections, primary nephrotic syndrome, Chinese medicine

## Abstract

**Background:**

Considering the adverse reactions and side effects of immunosuppressive and cytotoxic drugs for the treatment of Primary Nephrotic Syndrome (PNS) and the extensive exploration of Chinese herbal injections (CHIs), systematic evaluation of the efficacy of different CHIs in the treatment of PNS is a key imperative. In this study, we performed a network meta-analysis to investigate the efficacy of CHIs in the treatment of PNS.

**Methods:**

A systematic literature review including studies published from the establishment of each database to May 28, 2020, was conducted in PubMed, the Cochrane Library, Embase, Web of Science, the Chinese Biological Medicine Literature Service System (CBM), the China National Knowledge Infrastructure (CNKI) database, the Chinese Scientific Journal Database (VIP), and the Wanfang Database (WF).Two evaluators independently screened the literature, extracted data and the Cochrane Reviewer’s Handbook 5.1 method was used to evaluate the quality of included studies. The differences in efficacy of different CHIs were compared and ranked using Stata 16.0 software. Surface under the cumulative ranking curve (SUCRA) probability values were applied to rank the examined treatments. Clustering analysis was performed to compare the effects of CHIs between two different outcomes.

**Results:**

A total of 41 eligible randomized controlled trials involving 2879 patients and nine CHIs were included. Nine CHIs were Xiangdan injection (XDI), Huangqi injection (HQI), Shenkang injection (SKI), Danshen injection (DSI), Yinxingdamo injection (YXI), Dengzhanhuasu injection (DZI), Danhong injection (DHI), Shuxuetong injection (SXI), Chuanxiongqin injection (CXI). The results of the network meta-analysis showed that: with Western medical (WM) treatment as a co-intervention, in terms of improving the total clinical effectiveness and serum albumin level, DHI was the most likely to be the best choice for treatment (SUCRA = 82.2%); YXI had the highest probability of being the best option in terms of reducing 24-h urinary protein excretion (SUCRA = 97.8%); in cholesterol-lowering comparisons, the SUCRA value allows for the most likely to be the best treatment is DZI (SUCRA = 84.5%). SXI was the most effective CHIs in terms of lowering serum triglycerides (SUCRA = 85.6%), whereas on the reducing fibrinogen side, the efficacy of CXI was significant (SUCRA = 67.6%). The result cluster analysis indicated that YXI and DHI were the best interventions with respect to total clinical effectiveness, 24-h urinary protein excretion and serum albumin.

**Conclusions:**

CHIs were found to be superior to WM alone in the treatment of PNS and may be beneficial for patients with PNS. WM+YXI and WM+DHI had the potential to be the best CHI with respect to the total clinical effectiveness, 24-h urinary protein excretion and serum albumin. However, more well-designed randomized controlled trials are still warranted.

## Introduction

Nephrotic syndrome (NS) is the pathologic condition of large amount of proteinuria (> 3.5 g/d), hypoalbuminemia (< 30 g/L), edema and/or hyperlipidemia caused by the loss of plasma protein in the urine due to the damage of the basement membrane of the glomerulus and the failure of the glomerular filtration barrier. Among them, primary nephrotic syndrome (PNS) means a type of nephrotic syndrome of unknown etiology, the mechanism of which is mostly mediated by immune inflammation. The main pathological types of PNS are microscopic nephropathy, mesenteric proliferative glomerulonephritis, mesenteric capillary glomerulonephritis, membranous nephropathy, and focal segmental glomerulosclerosis. The current mainstream medical treatment of PNS mainly uses glucocorticosteroid combined with immunosuppressive agents (Zhang J. et al., 2018). Recent years biologic agents have become a crucial treatment option in PNS. A meta-analysis showed that although rituximab has a beneficial effect and can reduce proteinuria on PNS, there are still some adverse events in the treatment process (Eckardt and Kasiske, 2009). Due to the lack of accurate understanding of its potential pathogenesis and causes, the treatment effect is not satisfactory, and most of patients with PNS are associated with thromboembolism and other adverse effects have resulted in some patients suffering from unnecessary toxic side effects from immunosuppressants (Liebeskind, 2014). In recent years, there have been increasing reports on the use of traditional Chinese medicine (TCM) in the treatment of PNS. TCM-assisted treatment of PNS has obvious advantages of not only improving the therapeutic effect, but also weakening the effect of glucocorticosteroid or immunosuppressive drugs’ toxic side effects to some extent (Wang et al., 2018; Deng et al., 2020). The treatment of PNS with Chinese medicine injections (CHIs) can reflect in multiple pathways and mechanisms, for example, the total flavonoids of astragalus in astragalus injection may play a role in treating nephrotic syndrome by regulating signal pathways such as AGE-RAGE, PI3K/Akt, VEGF, IL-17, and MAPK (Zhang et al., 2018). CHIs are sterile preparations made from chinese herbal medicines after extraction and purification for input into the human body, which is a combination of traditional medicine theory and modern medicine. It has the advantages of high bioavailability and fast onset of action, and has widely used in clinical applications, but there are more varieties available in CHIs. Differences in clinical efficacy and safety are also unclear, thus creating confusion for patients and physicians. Network meta-analysis allows multiple interventions to be compared and ranked for efficacy or safety to select the best ones (De Laat, 2017). Therefore, this study adopts this method to evaluate the efficacy and safety of various CHIs for the adjuvant treatment of adult PNS, with a view to provide evidence-based medical evidences supporting for the selection of CHIs adjuvant treatment of PNS in clinical practice. This study is reported in strict accordance with the standard format of the Preferred Reporting Items for Systematic Reviews and Meta-Analysis Specification: PRISMA Extension Statement specification (Moher et al., 2010).

## Information and Methods

This systematic review has been registered in International Platform of Registered Systematic Review and Meta-Analysis Protocols (INPLASY). The registration number is INPLASY202080091. It followed the Preferred Reporting Items for Systematic Review and Meta- Analysis (PRISMA) available at the attachment 2.

### Inclusion and Exclusion Criteria

i. Study type: all published randomized controlled trials (RCT) or controlled clinical trials (CCT), in Chinese and English only; ii. Subjects: The subjects of the study are those who meet the requirements of the PNS diagnostic criteria. age, gender, disease duration, race, and region are not limited; iii. Interventions: the treatment group adopts traditional CHIs in combination with conventional western medicine (WM), the control group was treated with another CHIs in combination with WM or with WM alone whereas WM treatment needs to be consistent between treatment group and control group; iv. Outcomes: The primary outcomes in this article were total clinical effectiveness (TER), 24-h urinary protein excretion (24h-UTP), serum albumin (ALB); and the secondary outcomes were cholesterol (TC), triglycerides (TG), fibrinogen (Fib), Security evaluation, the literatures including one primary outcomes is sufficient; v. Exclusion criteria: duplicate publications; inaccessible literature; inaccessible data extraction of studies; studies in which the intervention was a combination of two or more CHIs were excluded; studies in which the evaluation indicators did not include.

### Search Strategy

**Figure 1:**
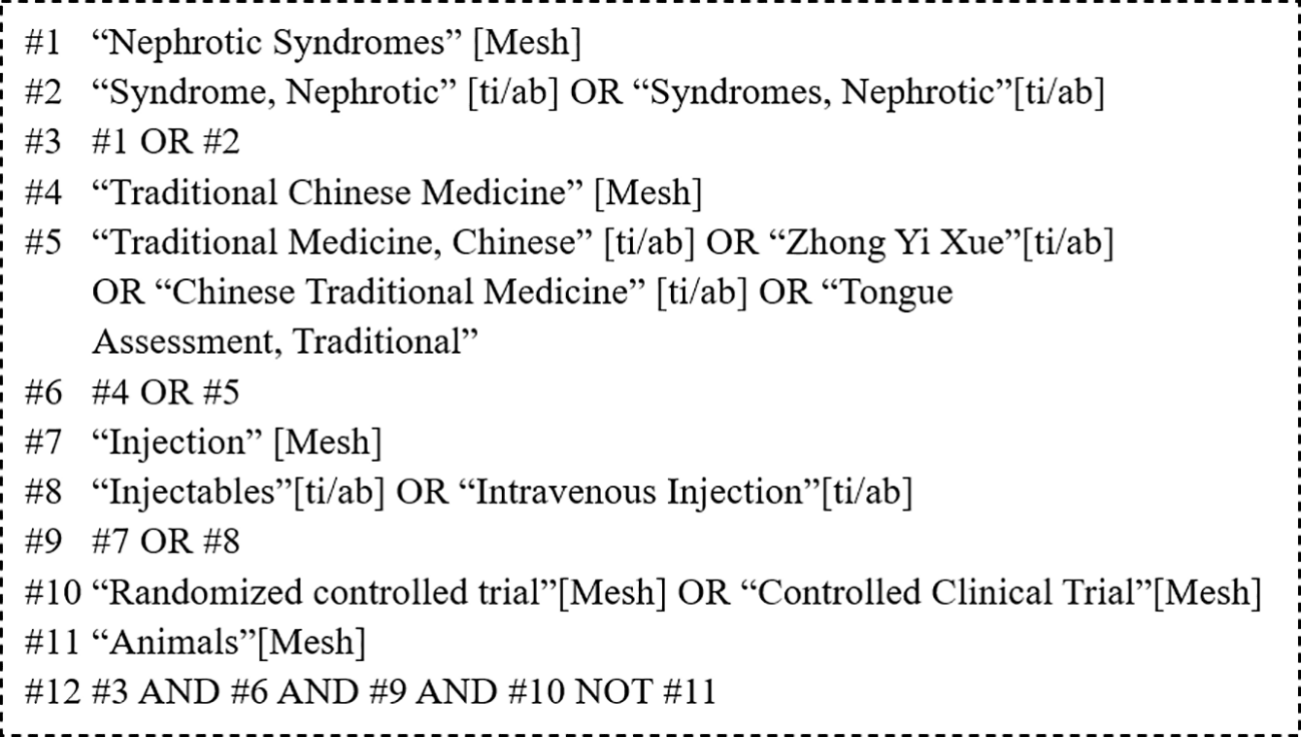
The specific search terms of PubMed.

PubMed, the Cochrane Library, Embase, Web of Science, the Chinese Biological Medicine Literature Service System (CBM), the China National Knowledge Infrastructure (CNKI) database, the Chinese Scientific Journal Database (VIP), and the Wanfang Database (WF) were searched for RCTs of CHIs for the treatment of PNS. Studies published from the establishment of each database to May 28, 2020 were eligible for inclusion. In addition, the reference lists of the included studies were manually searched to identify relevant literature to make the research information more comprehensive. There were three parts of the search strategy, including primary nephrotic syndrome, chinese herbal injection, and random controlled trial. The specific search terms of PubMed are shown in **Figure 1**.

### Literature Inclusion and Data Extraction

We used Endnote software to manage all retrieved studies. After excluding duplicates, two researchers independently screened the retrieved studies based on the inclusion and exclusion criteria and extracted the data from the included RCTs. Then, read literature titles and abstracts for primary screening; and then full-text acquisition and reading of the literature for re-screening. The inclusion process was done independently by each of the 2 researchers. If the results were confused, and controversial, the group will consult third researcher. Relevant information was extracted which included: basic patient information, baseline status, intervention measures and course of treatment, and outcomes, etc. finally.

### Quality Assessment

2 researchers independently used the risk of bias assessment tool recommended by the Cochrane Systematic Evaluator’s Handbook 5.3 to assess the included studies. The researchers cross-checked the study independently and if there was any disagreement, it was resolved through discussion or with the assistance of a 3rd researcher.

### Statistical Analysis

The Total Clinical Effectiveness is the count data, so the odds ratio (OR) and its 95% CI are used. As well we used the mean difference (Mean Difference, MD) and its 95% CI to calculate Measuring data, such as Ending indicators 24h-UTP, ALB, etc. Direct comparison of heterogeneity between studies using χ 2 test for analysis (test level of α = 0.05). The inconsistency factor (IF) and the Z-test P value were used to determine the consistency of the results of direct and indirect comparisons, if P > 0.05 and IF Smaller values indicate better consistency. According to the cumulative ranking probability curve (surface under the cumulative ranking area, SUCRA) to rank the intervention effects. If the number of studies was ≥10, funnel plots were plotted to identify the presence of publication bias. Plotting comparison-corrected funnel plots to assess small-sample effects; if the funnel plot scatter was roughly symmetrical, a small-sample effect was considered absent. The opposite is considered to exist. In addition cluster analysis attempts to suggest the best intervention for PNS. All the statistical results and statistical graphing of the study were done using Stata 16.0 software.

## Results

**Figure 2:**
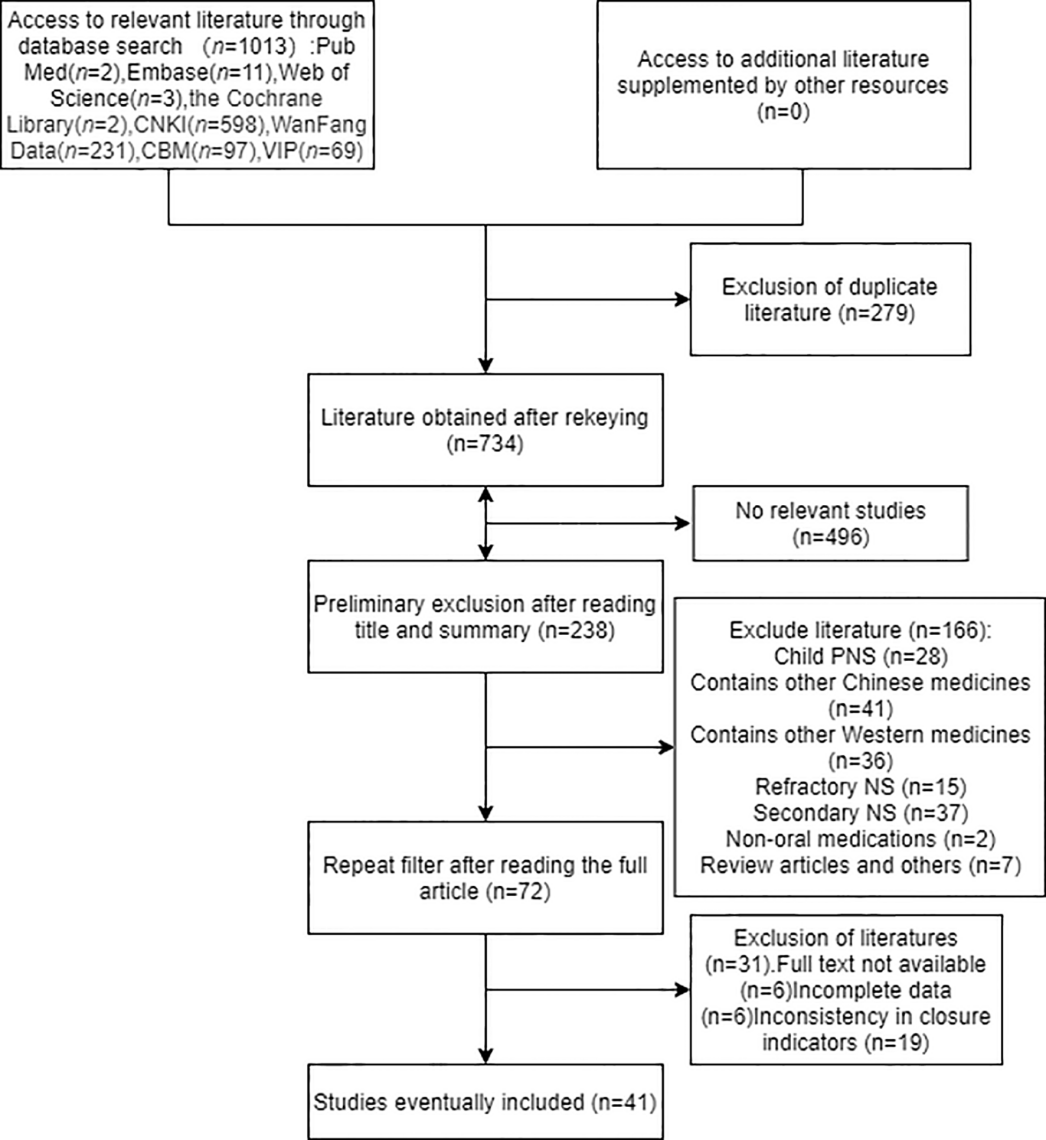
Literature screening map.

1013 records were initially retrieved, including 598 articles in CNKI, 231 in WF, 69 in VIP, 97 in CBM, and 2 articles in PubMed, 11 in Embase, 3 articles in Web of Science, 2 articles in The Cochrane Library. Manual search and reference tracing did not find any eligible literature. After deduplicating articles as well as reading the abstracts and eliminating unqualified literatures, 41 eligible studies were identified. Further details of the literature screening process are shown in **Figure 2**.

### Characteristics of the Studies Included

**Figure 3:**
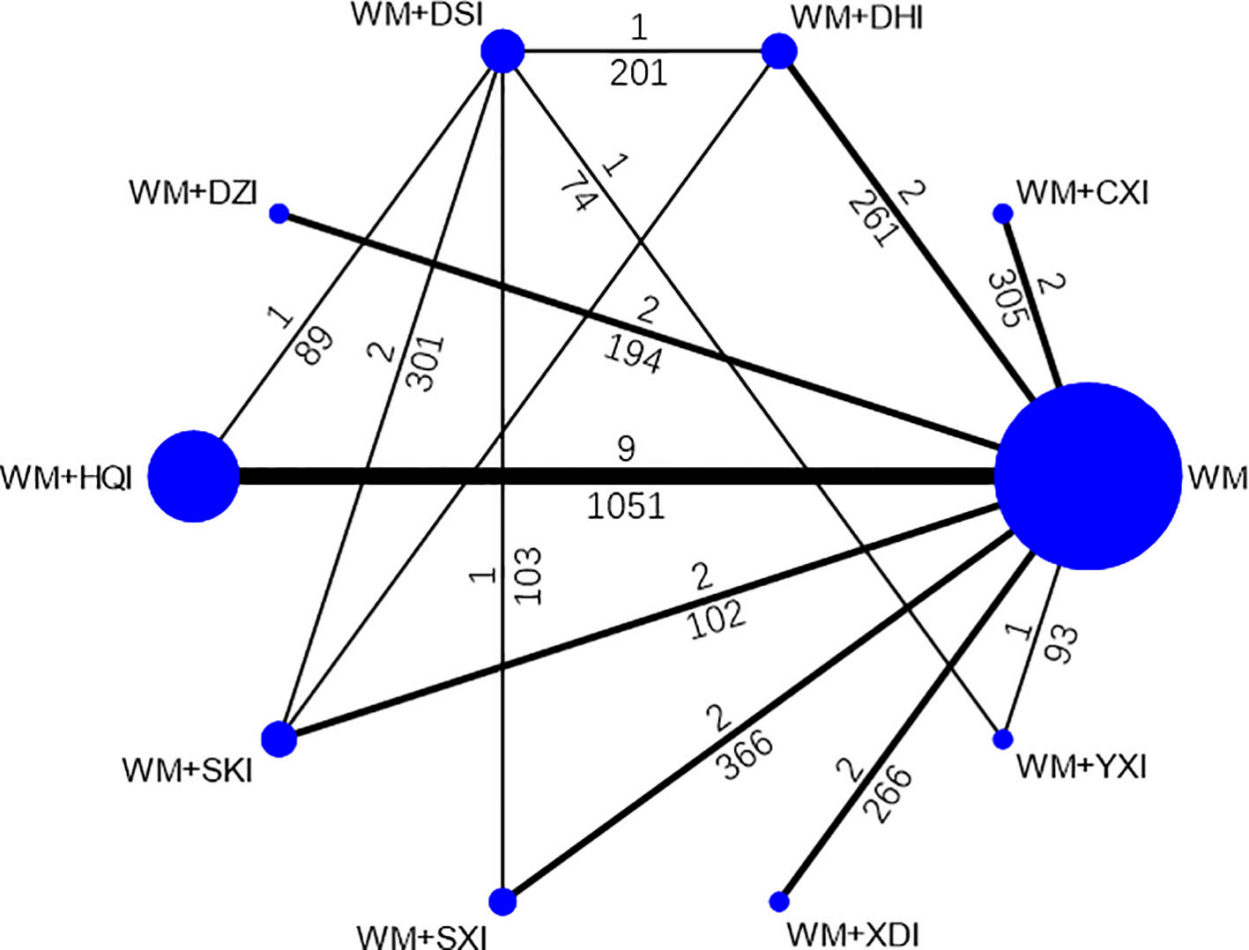
Network graph for total clinical effectiveness. WM, Western medicine; XDI, Xiangdan injection; HQI, Huangqi injection; SKI, Shenkang injection; DSI, Danshen injection; YXI, Yinxingdamo injection; DZI, Dengzhanhuasu injection; DHI, Danhong injection; SXI, Shuxuetong injection; CXI, Chuanxiongqin injection.

41 included studies (Zhuang, 1998; Lin and Lai, 1999; Ma and Cheng, 2002; Shen et al., 2002; Yang et al., 2002; Zhang, 2003; Bai, 2004; Niu and Niu, 2004; Ye and Zhang, 2006; Deng, 2007; Li, 2007; Liu, 2008; Liu and Zhu, 2008; Xu, 2008; Dai and Zhang, 2009; Xianrong and Bangcui, 2009; Yuan and Tao, 2009; Yuan et al., 2009; Feng et al., 2010; Song and Zhang, 2010; Tursun and Liang, 2010; Yang et al., 2010; Chu et al., 2011; Li, 2011; Xie et al., 2011; Yang et al., 2011; Zhang, 2011; Zhang et al., 2011; Lei and Hu, 2012; Wang, 2012; Cai and Zhu, 2013; Li and Xu, 2013; Fu and Zhang, 2014; Gong, 2014; Liu, 2014; Chen et al., 2015; Sun, 2015; Li et al., 2016; Zhang and Bian, 2016; Li and Wang, 2017; Long, 2018) involved 2,879 patients with PNS, 1411 and 1468 in the treatment and control groups, respectively, and the course of treatment was 5 days to 6 months. All of the publications were two-arm studies involving nine herbal injections, namely, Xiangdan injection (XDI)(2 articles), Huangqi injection (HQI 15 articles), Shenkang injection (SKI 3 articles), Danshen injection (DSI 12 articles), Yinxingdamo injection (YXI 5 articles), Dengzhanhuasu injection (DZI 3 articles), Danhong injection (DHI 3 articles), Shuxuetong injection (SXI 5 articles), and Chuanxiongqin injection (CXI 3 articles). The control interventions were western basal therapeutic ways and some immunosuppressants such as valsartan, benazepril, methylprednisolone, and cyclophosphamide, morpholipid, etc. Of the 41 included documents, 28 (Zhuang, 1998; Lin and Lai, 1999; Ma and Cheng, 2002; Yang et al., 2002; Bai, 2004; Ye and Zhang, 2006; Deng, 2007; Liu and Zhu, 2008; Liu, 2008; Xu, 2008; Xianrong and Bangcui, 2009; Yuan and Tao, 2009; Feng et al., 2010; Song and Zhang, 2010; Tursun and Liang, 2010; Yang et al., 2010; Chu et al., 2011; Xie et al., 2011; Yang et al., 2011; Zhang, 2011; Lei and Hu, 2012; Fu and Zhang, 2014; Liu, 2014; Sun, 2015; Li et al., 2016; Zhang and Bian, 2016; Li and Wang, 2017; Long, 2018) reported total clinical effectiveness and 35 (Zhuang, 1998; Lin and Lai, 1999; Ma and Cheng, 2002; Shen et al., 2002; Yang et al., 2002; Zhang, 2003; Bai, 2004; Niu and Niu, 2004; Ye and Zhang, 2006; Deng, 2007; Li, 2007; Liu, 2008; Liu and Zhu, 2008; Dai and Zhang, 2009; Yuan et al., 2009; Feng et al., 2010; Song and Zhang, 2010; Tursun and Liang, 2010; Chu et al., 2011; Li, 2011; Xie et al., 2011; Yang et al., 2011; Zhang, 2011; Zhang et al., 2011; Wang, 2012; Li and Xu, 2013; Fu and Zhang, 2014; Gong, 2014; Liu, 2014; Chen et al., 2015; Sun, 2015; Li et al., 2016; Zhang and Bian, 2016; Li and Wang, 2017; Long, 2018) reported 24-h urine protein, 29 papers (Zhuang, 1998; Lin and Lai, 1999; Shen et al., 2002; Yang et al., 2002; Zhang, 2003; Bai, 2004; Niu and Niu, 2004; Liu, 2008; Liu and Zhu, 2008; Dai and Zhang, 2009; Yuan et al., 2009; Feng et al., 2010; Song and Zhang, 2010; Tursun and Liang, 2010; Chu et al., 2011; Li, 2011; Xie et al., 2011; Yang et al., 2011; Zhang, 2011; Zhang et al., 2011; Wang, 2012; Li and Xu, 2013; Gong, 2014; Liu, 2014; Chen et al., 2015; Sun, 2015; Li et al., 2016; Zhang and Bian, 2016; Li and Wang, 2017) reported serum albumin levels, 26 papers (Zhuang, 1998; Ma and Cheng, 2002; Bai, 2004; Niu and Niu, 2004; Ye and Zhang, 2006; Deng, 2007; Li, 2007; Liu, 2008; Dai and Zhang, 2009; Yuan and Tao, 2009; Yuan et al., 2009; Feng et al., 2010; Song and Zhang, 2010; Tursun and Liang, 2010; Chu et al., 2011; Yang et al., 2011; Zhang et al., 2011; Zhang, 2011; Wang, 2012; Cai and Zhu, 2013; Li and Xu, 2013; Gong, 2014; Sun, 2015; Li et al., 2016; Zhang and Bian, 2016; Long, 2018) reported cholesterol, triglyceride levels were reported in 22 papers (Ma and Cheng, 2002; Bai, 2004; Niu and Niu, 2004; Ye and Zhang, 2006; Deng, 2007; Li, 2007; Liu, 2008; Dai and Zhang, 2009; Yuan and Tao, 2009; Yuan et al., 2009; Song and Zhang, 2010; Tursun and Liang, 2010; Chu et al., 2011; Yang et al., 2011; Zhang, 2011; Zhang et al., 2011; Wang, 2012; Cai and Zhu, 2013; Li and Xu, 2013; Gong, 2014; Sun, 2015; Zhang and Bian, 2016), and fibrinogen was reported in 17 papers (Zhuang, 1998; Niu and Niu, 2004; Ye and Zhang, 2006; Deng, 2007; Li, 2007; Liu, 2008; Dai and Zhang, 2009; Yuan and Tao, 2009; Yuan et al., 2009; Song and Zhang, 2010; Yang et al., 2010; Chu et al., 2011; Zhang et al., 2011; Wang, 2012; Li and Xu, 2013; Li et al., 2016; Li and Wang, 2017). The details of the included studies are shown in **Table 1**. Network graph for total clinical effectiveness is shown in **Figure 3**.

**Table 1:** Characteristics of the studies included in this meta-analysis.

Study ID	Cases (T/C)	Age (T/C)	Sex(M/F)	course of disease (T/C)	Interventions		Course	Outcomes
					T	C		
Ye and Zhang, 2006	36/20	42∼16/38∼14	34/22	30 d∼2 y	BM+XDI	BM	30 d	①③④⑤⑥
Li and Wang, 2017	42/42	37.5 ± 4.2/35.8 ± 3.7	38/46	11.9 ± 3.2 m/ 12.4 ± 4.1 m	BM+XDI	BM	5–10 d	①②③
Sun, 2015	48/48	48.2 ± 3.4/41.9 ± 3.2	57/39	–	BM+HQI	BM	1 m	①②③④⑤
Chen et al., 2015	60/60	23.1 ± 1.1/22.0 ± 1.3	71/49	–	BM+HQI	BM	–	②③
Liu, 2014	20/20	18∼58/14∼61	24/16	–	BM+HQI	BM	1 m	①②③⑦
Gong, 2014	32/32	18∼60	29/35	–	BM+HQI	BM	1 m	②③④⑤
Fu and Zhang, 2014	30/30	20 ± 3.5/18 ± 4.3	25/35	3–25 d/5–16 d	BM+SKI	BM+DSI	14 d	①②⑦
Li and Xu, 2013	25/25	14∼52/12∼50	37/13	10–1.5 y/11–1.8 y	BM+YXI	BM+DSI	4 w	②③④⑤⑥
Cai and Zhu, 2013	28/28	34∼69/35∼72	31/25	–	BM+DZI	BM	2 w	④⑤⑦
Wang, 2012	120/118	16∼57	141/97	–	BM+YXI	BM	6 w	②③④⑤⑥⑦
Lei and Hu, 2012	21/18	16∼48/14∼53	13/26	–	BM+HQI	BM	–	①
Zhang et al., 2011	30/30	14∼52/12∼50	40/20	7 d–1.5 y/7 d–2 y	BM+YXI	BM+DSI	14 d	②③④⑤⑥
Zhang, 2011	30/30	35.8 ± 12.4	28/32	0.5 m–5 y	BM+DZI	BM	2 w	①②③④⑤⑦
Yang et al., 2011	30/26	39.6 ± 12.8/38.8 ± 13.5	31/25	3.4 ± 0.6∼/3.3 ± 0.3 y	BM+DHI	BM	4 m	①②③④⑤
Xie et al., 2011	50/50	38.6 ± 11.5/39.4 ± 11.6	70/30	11 d–12 m/10 d–12 m	BM+SKI	BM+DSI	4 w	①②③⑦
Li, 2011	18/21	23.5 ± 5.2/21.3 ± 6.9	25/14	∼	BM+HQI	BM	6 w	②③
Yang et al., 2010	44/44	17∼56/20∼50	50/38	1–12 m/2–13 m	BM+ CTX +DHI	BM+ CTX	–	①⑥
Song and Zhang, 2010	28/30	30 ± 9.9/ 29.93 ± 9.15	31/27	8.68 ± 6.14/7.97 ± 6.37 m	BM+SXI	BM+DSI	2 m	①②③④⑤⑥
Zhou and Fu, 2009	40/40	15∼45	61/19	–	BM+HQI	BM	14–30 d	①
Yuan and Tao, 2009	38/36	36∼69/35∼70	35/39	–	BM+SXI	BM	3 w	①④⑤⑥
Yuan et al., 2009	26/22	30 ± 2.4/34.2 ± 3.9	24/22	–	BM+DSI	BM	28 d	②③④⑤⑥
Dai and Zhang, 2009	33/33	18∼65/18∼63	45/21	3 m–4 y	BM+CXI	BM	3 w	②③④⑤⑥⑦
Xu, 2008	19/21	20 ± 3	25/15	–	BM+HQI	BM	14–30 d	①
Liu and Zhu, 2008	40/40	24.8 ± 11.7/23.8 ± 12	58/32	0.6–13 y/0.–14 y	BM+ CTX +HQI	BM+ CTX	45 d	①②③⑦
Tursun and Liang, 2010	32/20	33 ± 4/34 ± 4	29/23	0.5 m–6 y	BM+YXI	BM	6 m	①②③④⑤⑦
Li, 2007	56/60	30 ± 9.9/ 29.93 ± 9.15	62/54	8.58 ± 6.14/7.97 ± 6.37 m	BM+SXI	BM+DSI	2 m	②④⑤⑥
Deng, 2007	30/30	18∼66	30/30	1–6 m	BM+YXI	BM+DSI	4 w	①②④⑤⑥
Chu et al., 2011	68/62	17 ± 3/16 ± 3	78/52	–	BM+SYI	BM	15–30 d	①②③④⑤⑥⑦
Ma and Cheng, 2002	26/21	17∼56/15∼55	27/20	4 m	BM+DSI	BM	28 d	①②④⑤
Zhang and Bian, 2016	45/45	35.2 ± 10.1/35.3 ± 10.2	57/43	–	BM+HQI	BM	4 w	①②③
Liu, 2008	26/22	14∼64	23/25	–	BM+HQI	BM+DSI	4 w	①②③④⑤⑥
Niu and Niu, 2004	30/26	16∼62	32/24	–	BM+SXI	BM+DSI	2 w	②③④⑤⑥
Bai, 2004	31/32	14∼57/14∼63	37/26	–	BM+HQI	BM	8 w	②③④⑤
Zhang, 2003	10/10	14∼47/17∼48	15/5	–	BM+DSI	BM	14 d	②③
Yang et al., 2002	19/19	15∼60∼	22/16	–	BM+HQI	BM	14 d	①②③⑦
Shen et al., 2002	28/28	17∼51	32/24	–	BM+HQI	BM	10 w	②③
Lin and Lai, 1999	20/20	16∼67/17∼65	28/12	–	BM+HQI	BM	4 w	①②③⑦
Long, 2018	16/14	36.8 ± 3.6/37.0 ± 3.5	19/11	2.1 ± 0.7 y/2 ± 0.8 y	BM+CTX+SKI	BM+CTX	3 m	①②④
Zhuang, 1998	25/20	16∼52/14∼49	32/14	–	BM+CXI	BM	4–6 w	①②③④⑥
Li et al., 2016	58/58	27.45 ± 4.63/27.47 ± 4.65	59/57	6.63 ± 1.27 m/6.65 ± 1.29 m	BM+CXI	BM+MMF	14 d	①②③④⑥⑦
Feng et al., 2010	60/60	27.32 ± 10.33/32.23 ± 15.77	61/59	–	BM+DSI	BM+DHI	4 w	①②③④⑦

BM, Western basal therapeutic ways; XDI, Xiangdan injection; HQI, Huangqi injection; SKI, Shenkang injection; DSI, Danshen injection; YXI, Yinxingdamo injection; DZI, Dengzhanhuasu injection; DHI, Danhong injection; SXI, Shuxuetong injection; CXI, Chuanxiongqin injection; BM, Basic Treatment of Western medicine; CTX, Cyclophosphamide; MMF, Mycophenolate Mofetil; ①, Total Clinical Effectiveness; ②, 24-h urinary protein excretion; ③, Serum albumin; ④, Cholesterol; ⑤, Triglycerides; ⑥, Fibrinogen; ⑦, Safety index;d, day; m, month; y, year.

### Risk of Bias Assessment

**Figure 4:**
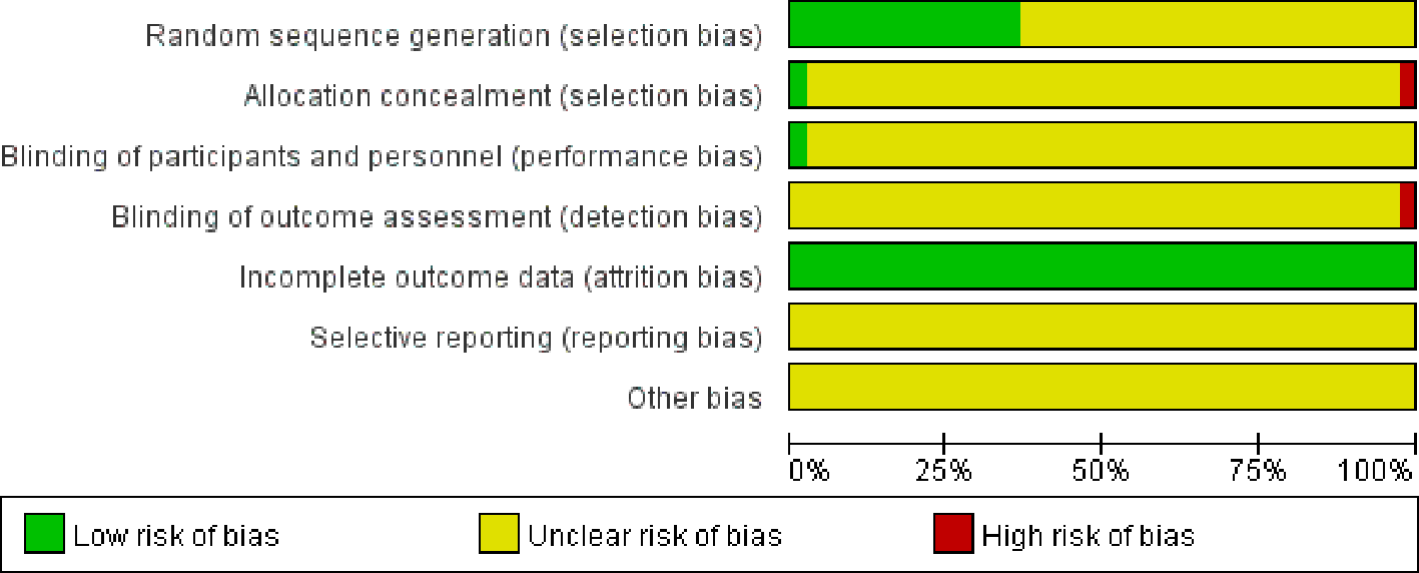
Summary of the risk of bias.

41 studies, all had references to randomization and only 5 papers (Ma and Cheng, 2002; Niu and Niu, 2004; Xianrong and Bangcui, 2009; Feng et al., 2010; Cai and Zhu, 2013) specifically reported cases random assignment method, mainly random number table method, semi-random assignment by order of visit/admission; all but 2 of 41 literatures (Ma and Cheng, 2002; Cai and Zhu, 2013) reported the application of blinding except for the rest of the included articles. All included studies outcome information was completed and other sources of bias could not be judged. In summary, the quality of included RCTs was poor (**Figure 4**).

### Results of the Network Meta-Analysis

#### Total Clinical Effectiveness (TCE)

For the sake of convenience, we have combined the complete remission rate with the partial remission rate. 28 of all 41 studies (Zhuang, 1998; Lin and Lai, 1999; Ma and Cheng, 2002; Yang et al., 2002; Bai, 2004; Ye and Zhang, 2006; Deng, 2007; Xu, 2008; Liu, 2008; Liu and Zhu, 2008; Xianrong and Bangcui, 2009; Yuan and Tao, 2009; Feng et al., 2010; Song and Zhang, 2010; Tursun and Liang, 2010; Yang et al., 2010; Chu et al., 2011; Xie et al., 2011; Yang et al., 2011; Zhang, 2011; Lei and Hu, 2012; Fu and Zhang, 2014; Liu, 2014; Sun, 2015; Li et al., 2016; Zhang and Bian, 2016; Li and Wang, 2017; Long, 2018), including 9 CHIs and 1890 patients, reported Clinical remission rate measures outcomes in patients with PNS. Network meta-analysis results indicated that all 9 types of CHIs each significantly improved CR rate in PNS patients compared to WM alone, except for the groups receiving WM+XDI (OR = 0.94,95%CI [0.39,2.24]) or WM+ DZI (OR = 0.42, 95% CI [0.13,1.36]). In addition, WM +XDI was significantly inferior to WM + HQI (OR = 1.03,95% CI [0.03,2.04]), WM + DSI (OR = 0.18,95% CI [0.04,0.83]), WM + YXI (OR = 0.30, 95% CI [0.11, 0.81]), WM+DHI (OR = 0.21,95% CI [0.07,0.61]), and WM + CXI (OR = 0.76,95% CI [0.09,1.43]) in increasing total clinical effectiveness (**Table 2**). According to the SUCRA values obtained from the probability ranking table (**Table 3**), it can be seen that in the comparison of clinical effectiveness, the ranking of the efficacy of the 9 CHIs is as follows: WM+DHI>WM+CXI>WM+DSI> WM+SXI>WM+YXI>WM+DZI>WM+SKI>WM+HQI>WM +XDI.

**Table 2:** Results of the network meta-analysis.

Outcomes	WM	WM+XDI	WM+HQI	WM+SKI	WM+DSI	WM+YXI	WM+DZI	WM+DHI	WM+SXI	WM+CXI
**TCE**	0.94(0.39,2.24)	WM+XDI								
	0.26(0.11,0.61)	0.28(0.11,0.67)	WM+HQI							
	0.25(0.07,0.92)	0.27(0.06,1.28)	0.98(0.21,4.60)	WM+SKI						
	0.17(0.05,0.60)	0.18(0.04,0.83)	0.64(0.14,2.99)	0.65(0.11,3.99)	WM+DSI					
	0.28(0.12,0.64)	0.30(0.11,0.81)	1.10(0.37,3.28)	1.11(0.24,5.11)	1.70(0.37,7.74)	WM+YXI				
	0.42(0.13,1.36)	0.45(0.12,1.69)	1.61(0.44,5.88)	1.64(0.29,9.36)	2.51(0.44,14.19)	1.47(0.36,5.94)	WM+DZI			
	0.20(0.06,0.62)	0.21(0.07,0.61)	0.76(0.21,2.71)	0.77(0.14,4.32)	1.18(0.21,6.54)	0.69(0.19,2.56)	0.47(0.10,2.26)	WM+DHI		
	0.26(0.07,0.96)	0.28(0.06,1.33)	1.00(0.21,4.76)	1.01(0.16,6.34)	1.55(0.25,9.61)	0.91(0.19,4.26)	0.62(0.11,3.59)	1.31(0.23,7.49)	WM+SXI	
	0.29(0.17,0.49)	0.31(0.12,0.80)	1.11(0.42,2.97)	1.13(0.28,4.54)	1.73(0.44,6.87)	1.02(0.39,2.65)	0.69(0.19,2.48)	1.47(0.43,5.03)	1.12(0.27,4.59)	WM+CXI
**24h-UTP**	WM									
	−0.07 (−0.63,0.49)	WM+XDI								
	0.96 (−0.06,1.99)	1.03(0.03,2.04)	WM+HQI							
	0.48 (−0.25,1.22)	0.55 (−0.37,1.48)	−0.48 (−1.74,0.78)	WM+SKI						
	0.12 (−0.72,0.96)	0.19 (−0.82,1.20)	−0.85 (−2.17,0.48)	−0.37 (−1.48,0.75)	WM+DSI					
	1.76(1.01,2.51)	1.83(1.19,2.47)	0.80 (−0.36,1.96)	1.28(0.23,2.33)	1.64(0.52,2.77)	WM+YXI				
	0.90(0.12,1.69)	0.98(0.24,1.71)	−0.06 (−1.26,1.14)	0.42 (−0.66,1.50)	0.79 (−0.36,1.94)	−0.86 (−1.80,0.09)	WM+DZI			
	1.04(0.39,1.69)	1.11(0.51,1.72)	0.08 (−1.04,1.20)	0.56 (−0.42,1.54)	0.93 (−0.14,1.99)	−0.72 (−1.56,0.12)	0.14 (−0.77,1.04)	WM+DHI		
	0.11 (−0.82,1.05)	0.18 (−0.91,1.28)	−0.85 (−2.24,0.54)	−0.37 (−1.56,0.82)	−0.00 (−1.26,1.25)	−1.65 (−2.85, −0.45)	−0.79 (−2.01,0.43)	−0.93 (−2.07,0.21)	WM+SXI	
	0.69(0.30,1.08)	0.76(0.09,1.43)	−0.28 (−1.36,0.81)	0.20 (−0.63,1.04)	0.57 (−0.36,1.50)	−1.07 (−1.91, −0.24)	−0.22 (−1.09,0.65)	−0.35 (−1.10,0.40)	0.57 (−0.43,1.58)	WM+CXI
**ALB**	WM									
	−1.08 (−3.13,0.97)	WM+XDI								
	−3.47 (−6.59, −0.35)	−2.39 (−5.45,0.67)	WM+HQI							
	−3.60 (−6.09,−1.12)	−2.52 (−5.77,0.73)	−0.13 (−4.13,3.87)	WM+SKI						
	−5.10 (−9.24, −0.96)	−4.02 (−8.64,0.61)	−1.63 (−6.82,3.56)	−1.50 (−6.33,3.34)	WM+DSI					
	−4.54 (−7.45, −1.63)	−3.46 (−6.15, −0.76)	−1.07 (−4.98,2.84)	−0.94 (−4.80,2.93)	0.56 (−4.50,5.62)	WM+YXI				
	−4.28 (−8.32, −0.24)	−3.20 (−6.68,0.28)	−0.81 (−5.44,3.82)	−0.68 (−5.44,4.08)	0.82 (−4.97,6.60)	0.26 (−4.14,4.66)	WM+DZI			
	−5.91 (−8.16, −3.65)	−4.83 (−7.25, −2.41)	−2.44 (−6.04,1.16)	−2.31 (−5.66,1.05)	−0.81 (−5.53,3.91)	−1.37 (−4.74,2.00)	−1.63 (−5.86,2.61)	WM+DHI		
	−4.94 (−8.70, −1.18)	−3.86 (−8.14,0.43)	−1.47 (−6.36, 3.42)	−1.34 (−5.85,3.17)	0.16 (−5.44,5.76)	−0.40 (−5.16,4.35)	−0.66 (−6.18,4.86)	0.97 (−3.42,5.35)	WM+SXI	
	−5.18 (−6.38, −3.97)	−4.10 (−6.34, −1.85)	−1.71 (−5.00,1.59)	−1.57 (−4.32,1.18)	−0.08 (−4.39,4.24)	−0.64 (−3.73,2.46)	−0.90 (−5.03,3.24)	0.73 (−1.77,3.23)	−0.24 (−4.19,3.71)	WM+CXI
**TC**	WM									
	0.39 (−0.49,1.27)	WM+XDI								
	0.89 (−0.49,2.28)	0.50 (−0.87,1.87)	WM+HQI							
	0.84 (−0.32,2.00)	0.44 (−1.00,1.89)	−0.06 (−1.85, 1.74)	WM+SKI						
	1.65(0.29,3.01)	1.26 (−0.36,2.88)	0.76 (−1.18,2.69)	0.81 (−0.97,2.60)	WM+DSI					
	1.17(0.17,2.17)	0.77 (−0.16,1.71)	0.27 (−1.29,1.84)	0.33 (−1.20,1.86)	−0.48 (−2.17,1.20)	WM+XYI				
	2.40(0.34,4.46)	2.01 (−0.23,4.24)	1.51 (−0.97,3.98)	1.56 (−0.80,3.92)	0.75 (−1.72,3.21)	1.23 (−1.06,3.52)	WM+DZI			
	1.27(0.29,2.24)	0.87 (−0.04,1.78)	0.37 (−1.18,1.92)	0.43 (−1.07,1.93)	−0.39 (−2.06,1.29)	0.10 (−1.10,1.29)	−1.13 (−3.41,1.14)	WM+DHI		
	1.28 (−0.78,3.34)	0.89 (−1.36,3.13)	0.39 (−2.10,2.87)	0.44 (−1.92,2.81)	−0.37 (−2.84,2.10)	0.11 (−2.18,2.41)	−1.12 (−4.03,1.79)	0.01 (−2.27,2.30)	WM+SXI	
	1.72(0.81,2.63)	1.32(0.15,2.50)	0.82 (−0.80,2.44)	0.88 (−0.60,2.36)	0.07 (−1.57,1.70)	0.55 (−0.75,1.85)	−0.68 (−2.93,1.57)	0.45 (−0.83,1.74)	0.44 (−1.82,2.69)	WM+CXI
**TG**	WM									
	0.31 (−0.15,0.76)	WM+XDI								
	0.31 (−0.64,1.26)	0.00 (−1.05,1.06)	WM+XDI							
	0.20 (−0.75,1.15)	−0.11 (−1.16,0.95)	−0.11 (−1.46,1.24)		WM+DSI					
	0.75(0.21,1.29)	0.44 (−0.26,1.15)	0.44 (−0.65,1.53)		0.55 (−0.55,1.64)	WM+XYI				
	0.89(0.40,1.39)	0.59(0.15,1.03)	0.58 (−0.48,1.65)		0.69 (−0.38,1.77)	0.15 (−0.58,0.87)	WM+DZI			
	0.57(0.09,1.05)	0.26 (−0.18,0.70)	0.26 (−0.80,1.32)		0.37 (−0.70,1.44)	−0.18 (−0.90,0.54)	−0.32 (−0.89,0.24)	WM+DHI		
	1.11(0.20,2.02)	0.80 (−0.21,1.82)	0.80 (−0.52,2.12)		0.91 (−0.41,2.23)	0.36 (−0.70,1.42)	0.22 (−0.82,1.25)	0.54 (−0.49,1.57)	WM+SXI	
	0.54(0.11,0.96)	0.23 (−0.38,0.84)	0.23 (−0.81,1.27)		0.34 (−0.71,1.38)	−0.21 (−0.90,0.47)	−0.36 (−1.00,0.28)	−0.03 (−0.66,0.59)	−0.57 (−1.58,0.43)	WM+CXI
**Fib**	WM									
	0.14 (−1.84,2.12)	WM+XDI								
	1.84 (−1.52,5.20)	1.70 (−2.19,5.60)	WM+HQI							
	1.08 (−0.92,3.08)	0.94 (−1.87,3.76)	−0.76 (−4.67,3.15)		WM+DSI					
	1.63 (−0.30,3.57)	1.49 (−0.22,3.21)	−0.21 (−4.08,3.67)		0.55 (−2.23,3.34)	WM+YXI				
	1.28 (−0.97,3.53)	1.14 (−0.64,2.93)	−0.56 (−4.60,3.48)		0.20 (−2.81,3.21)	−0.35 (−2.66,1.95)		WM+DHI		
	1.04 (−1.36,3.43)	0.90 (−2.21,4.00)	−0.80 (−4.92,3.32)		−0.04 (−3.16,3.08)	−0.60 (−3.67,2.48)		−0.24 (−3.53,3.04)	WM+SXI	
	1.94 (−2.01,5.88)	1.80 (−1.61,5.21)	0.10 (−5.08,5.27)		0.86 (−3.56,5.28)	0.31 (−3.51,4.12)		0.66 (−3.19,4.50)	0.90 (−3.71,5.51)	WM+CXI

WM, Western medicine; XDI, Xiangdan injection; HQI, Huangqi injection; SKI, Shenkang injection; DSI, Danshen injection; YXI, Yinxingdamo injection; DZI, Dengzhanhuasu injection; DHI, Danhong injection; SXI, Shuxuetong injection; CXI, Chuanxiongqin injection.

**Table 3:** Ranking of the efficacy of various interventions to treat PNS.

Outcomes	Interventions	SUCRA (%)	Mean rank
TCE	WM	0.0	9.8
	WM+XDI	12.2	8.9
	WM+HQI	42.6	6.2
	WM+SKI	43.9	6.0
	WM+DSI	66.6	4.0
	WM+YXI	59.2	4.7
	WM+DZI	55.5	5.0
	WM+DHI	82.2	2.6
	WM+SXI	64.8	4.2
	WM+CXI	70.8	3.6
24h-UTP	WM	17	8.5
	WM+XDI	14.4	8.7
	WM+HQI	69.9	3.7
	WM+SKI	46.3	5.8
	WM+DSI	25.5	7.7
	WM+YXI	97.8	1.2
	WM+DZI	68.2	3.9
	WM+DHI	75.6	3.2
	WM+SXI	27.1	7.6
	WM+CXI	58.1	4.8
ALB	WM	2.3	9.8
	WM+XDI	12.2	8.9
	WM+HQI	42.6	6.2
	WM+SKI	43.9	6.0
	WM+DSI	66.6	4.0
	WM+YXI	59.2	4.7
	WM+DZI	55.5	5.0
	WM+DHI	82.2	2.6
	WM+SXI	64.8	4.2
	WM+CXI	70.8	3.6
TC	WM	5.7	9.5
	WM+XDI	19.3	8.3
	WM+HQI	41.8	6.2
	WM+SKI	38.7	6.5
	WM+DSI	69.3	3.8
	WM+YXI	53.0	5.2
	WM+DZI	84.5	2.4
	WM+DHI	57.6	4.8
	WM+SXI	55.1	5.0
	WM+CXI	74.9	3.3
TG	WM	9.1	8.3
	WM+XDI	31.4	6.5
	WM+HQI	36.5	6.1
	WM+DSI	29.9	6.6
	WM+YXI	68.7	3.5
	WM+DZI	81.0	2.5
	WM+DHI	55.8	4.5
	WM+SXI	85.6	2.2
	WM+CXI	52.0	4.8
Fib	WM	18.2	6.7
	WM+XDI	22.4	6.4
	WM+HQI	66.1	3.4
	WM+DSI	51.8	4.4
	WM+YXI	67.0	3.3
	WM+DHI	57.0	4.0
	WM+SXI	49.9	4.5
	WM+CXI	67.6	3.3

WM, Western medicine; XDI, Xiangdan injection; HQI, Huangqi injection; SKI, Shenkang injection; DSI, Danshen injection; YXI, Yinxingdamo injection; DZI, Dengzhanhuasu injection; DHI, Danhong injection; SXI, Shuxuetong injection; CXI, Chuanxiongqin injection.

#### 24-h Urinary Protein Excretion (24h-UTP)

**Figure 5:**
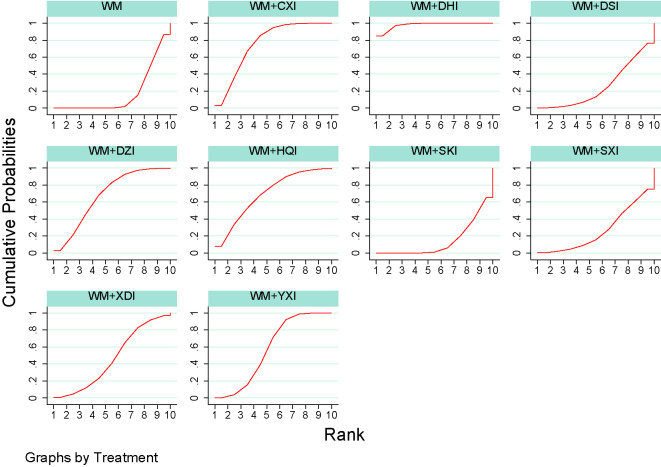
Plot of the surface under the cumulative ranking curves for all treatments in reducing 24h-UTP. WM, Western medicine; XDI, Xiangdan injection; HQI, Huangqi injection; SKI, Shenkang injection; DSI, Danshen injection; YXI, Yinxingdamo injection; DZI, Dengzhanhuasu injection; DHI, Danhong injection; SXI, Shuxuetong injection; CXI, Chuanxiongqin injection.

35 publications (Zhuang, 1998; Lin and Lai, 1999; Ma and Cheng, 2002; Shen et al., 2002; Yang et al., 2002; Zhang, 2003; Bai, 2004; Niu and Niu, 2004; Ye and Zhang, 2006; Deng, 2007; Li, 2007; Liu, 2008; Liu and Zhu, 2008; Dai and Zhang, 2009; Yuan et al., 2009; Feng et al., 2010; Song and Zhang, 2010; Tursun and Liang, 2010; Chu et al., 2011; Li, 2011; Xie et al., 2011; Yang et al., 2011; Zhang et al., 2011; Zhang, 2011; Wang, 2012; Li and Xu, 2013; Fu and Zhang, 2014; Gong, 2014; Liu, 2014; Chen et al., 2015; Sun, 2015; Li et al., 2016; Zhang and Bian, 2016; Li and Wang, 2017; Long, 2018) reported 24-h urinary protein excretion, including 2506 patients and 9 herbal injections. The network meta-analysis results showed that, there were no significant differences with Western medicine alone in the results including WM + XDI (MD = −0.07,95%CI[−0.63,0.49]), WM + HQI (MD = 0.96, 95%CI [−0.06,1.99]), and WM + SKI (MD = 0.48,95%CI [−0.25,1.22]). The remaining CHIs combined with western medicine were superior to western medicine alone, and the difference was statistically significant. 2e group receiving WM +XDI had shown significantly higher urinary protein excretion than those receiving WM+ HQI(MD = 0.28,95%CI[0.11,0.67), WM + YXI (MD = 1.83,95% CI[1.19,2.47]), WM + DZI (MD = 0.98, 95% CI [0.24, 1.71]), WM + DHI (MD = 1.11,95% CI [0.51,1.72]), and WM + CXI (MD = 0.31, 95% CI [0.12,0.80]). The group receiving WM + YXI had significantly lower urinary protein excretion than those receiving WM + SKI (MD = 1.28, 95% CI [0.23, 2.33]) and M+DSI(MD = 1.64, 95%CI [0.52,2.77]), while had higher than the group receiving WM + SXI (MD = −1.65,95% CI [−2.85, −0.45]) and WM + CXI (MD = −1.07, 95% CI[−1.91, −0.24]). According to the SUCRA values obtained from the probability ranking table (**Table 3**, **Figure 5**), it can be seen that the 9 CHIs’ efficacy rankings in the comparison of reducing 24h-UTP were: WM + XYI > WM + DHI > WM +HQI > WM +DZI > WM + CXI > WM + SKI> WM + SXI > WM + DSI> WM+XDI.

#### Serum Albumin (ALB)

**Figure 6:**
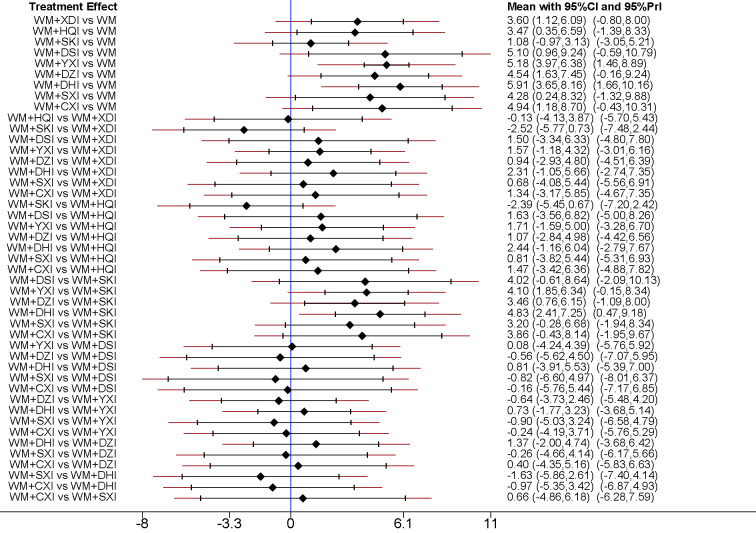
Forest plot of results of network meta-analysis on increasing ALB. WM, Western medicine; XDI, Xiangdan injection; HQI, Huangqi injection; SKI, Shenkang injection; DSI, Danshen injection; YXI, Yinxingdamo injection; DZI, Dengzhanhuasu injection; DHI, Danhong injection; SXI, Shuxuetong injection; CXI, Chuanxiongqin injection.

29 studies (Zhuang, 1998; Lin and Lai, 1999; Shen et al., 2002; Yang et al., 2002; Zhang, 2003; Bai, 2004; Niu and Niu, 2004; Liu, 2008; Liu and Zhu, 2008; Dai and Zhang, 2009; Yuan et al., 2009; Feng et al., 2010; Song and Zhang, 2010; Tursun and Liang, 2010; Chu et al., 2011; Li, 2011; Xie et al., 2011; Yang et al., 2011; Zhang, 2011; Zhang et al., 2011; Wang, 2012; Li and Xu, 2013; Gong, 2014; Liu, 2014; Chen et al., 2015; Sun, 2015; Li et al., 2016; Zhang and Bian, 2016; Li and Wang, 2017), involving 9 CHIs, reported serum albumin levels. The results of the network meta-analysis showed that all the groups of PNS patients receiving WM+CHIs had higher serum albumin levels than groups receiving WM alone except for WM+XDI, WM +HQI, WM + SKI. When compared to the group receiving WM+XDI, the groups receiving WM +HQI (MD = 0.28, 95%CI [0.11,0.67]), WM + XYI (MD = 1.83,95%CI[1.19,2.47]), WM +DZI (MD = 0.98, 95%CI[0.24,1.71]), WM + DHI (MD = 1.11, 95%CI[0.51,1.72]) or WM + CXI (MD = 0.31,95%CI[0.12,0.80]) showed significantly higher serum albumin levels. Additionally, groups of PNS patients receiving WM +YXI had significantly higher serum albumin levels than the group receiving WM+SHI (MD = 1.28,95%CI[0.23,2.33]) or WM+DSI (MD = 1.64,95%CI[0.52,2.77])WM+DSI while had significantly lower than the group receiving WM+SXI (MD = −1.65,95%CI[−2.85, −0.45])or WM+SXI (MD = −1.07,95%CI[−1.91, −0.24]) (**Table 2**, **Figure 6**). According to the SUCRA values obtained from the probability ranking table (**Table 3**), in the comparison of increasing in ALB levels, the order of nine CHIs is: WM+DHI>WM+CXI>WM+DSI>WM+SXI>WM+YXI>WM+DZI>WM+SKI>WM+HQI>WM +XDI.

#### Cholesterol (TC)

26 RCTs (Zhuang, 1998; Ma and Cheng, 2002; Bai, 2004; Niu and Niu, 2004; Ye and Zhang, 2006; Deng, 2007; Li, 2007; Liu, 2008; Dai and Zhang, 2009; Yuan and Tao, 2009; Yuan et al., 2009; Feng et al., 2010; Song and Zhang, 2010; Tursun and Liang, 2010; Chu et al., 2011; Yang et al., 2011; Zhang, 2011; Zhang et al., 2011; Wang, 2012; Cai and Zhu, 2013; Li and Xu, 2013; Gong, 2014; Sun, 2015; Li et al., 2016; Zhang and Bian, 2016; Long, 2018) addressed cholesterol outcomes in patients with PNS, including 1955 patients and 9 CHIs. The results of the network meta-analysis indicated that all groups of patients receiving CHIs +WM had significantly lower Cholesterol level than groups receiving WM alone, except for the groups receiving WM+XDI (MD = 0.39,95%CI[−0.49,1.27]),WM+HQI (MD = 0.89,95%CI[−0.49,2.28] or WM+SXI (MD = 1.28,95%CI[−0.78,3.34. Additionally, when compared to the group receiving WM+XDI, the groups receiving WM+CXI(MD = 1.32,95%CI[0.15,2.50]) showed significantly decreasing cholesterol levels (**Table 2**). According to the SUCRA values obtained from the probability ranking table (**Table 3**), it can be seen that in the comparison of lower cholesterol levels. The order of 9CHIs is as follows: WM+ DZI> WM+ CXI> WM+ DSI> WM+ DHI> WM+ SXI> WM+ YXI> WM+ HQI> WM+SKI> WM + XDI.

#### Triglycerides (TG)

Due to inconsistency in the triglycerides data (P = 0.001), the pairwise meta-analysis is shown as main results. the results suggest that all groups of PNS patients receiving CHIs +WM had significantly lower triglycerides than groups receiving WM alone (MD,− 0.70 to − 1.92), except for the groups receiving WM+XDI (MD = 0.31,95%CI[−0.15,0.76]), WM+HQI (MD = 0.31,95%CI[−0.64,1.26])or WM+DSI (MD = 0.20, 95%CI[−0.75,1.15]) (**Table 2**). According to the SUCRA values obtained from the probability ranking table (**Table 3**), it can be seen that in the comparison of lower triglycerides. The order of 8 CHIs is as follows: WM + SXI > WM + DZI> WM + YXI> WM + DHI> WM +CXI> WM + HQI > WM + XDI > WM + DSI.

#### 2.3.6 Fibrinogen (Fib)

17 RCTs (Zhuang, 1998; Niu and Niu, 2004; Ye and Zhang, 2006; Deng, 2007; Li, 2007; Liu, 2008; Dai and Zhang, 2009; Yuan and Tao, 2009; Yuan et al., 2009; Song and Zhang, 2010; Yang et al., 2010; Chu et al., 2011; Zhang et al., 2011; Wang, 2012; Li and Xu, 2013; Li et al., 2016; Li and Wang, 2017), involving 7 CHIs, reported fibrinogen, the network meta-analysis showed that all the groups of PNS patients receiving WM+CHIs (WM+XDI,WM+HQI, WM+DSI, WM+YXI, WM+DHI, WM+SXI, WM+CXI) had lower serum albumin levels than groups receiving WM alone. There were no significant differences in any of the groups (**Table 3**). According to the SUCRA values obtained from the probability ranking table (**Table 3**), it can be seen that in the comparison of reduce fibrinogen. The order of 7 CHIs is: WM+CXI> WM+YXI>WM+HQI> WM+ DHI> WM + DSI > WM +SXI> WM + XDI.

#### Inconsistency Tests

**Figure 7:**
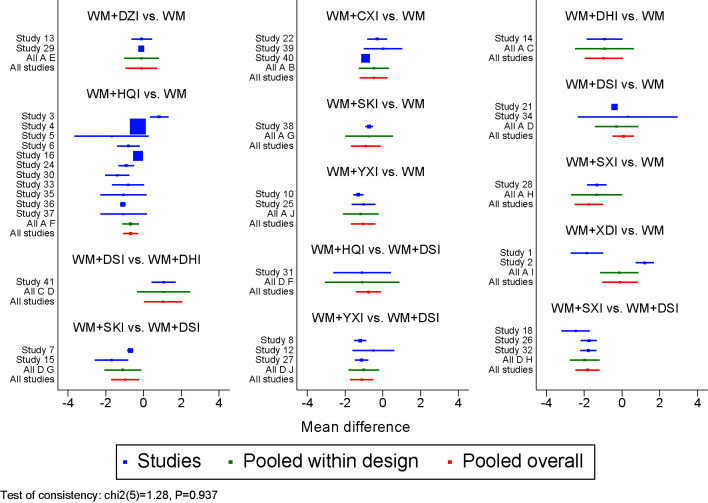
Forest plot of 24-UTP. WM, Western medicine; XDI, Xiangdan injection; HQI, Huangqi injection; SKI, Shenkang injection; DSI, Danshen injection; YXI, Yinxingdamo injection; DZI, Dengzhanhuasu injection; DHI, Danhong injection; SXI, Shuxuetong injection; CXI, Chuanxiongqin injection.

The results of the nodal analysis model showed P>0.05, suggesting that direct and indirect comparisons were consistent. However, several loops showed inconsistency for the outcomes of triglycerides, so pairwise meta- analysis were used as the main results for these two outcomes. The inconsistency test of the 24-UTP excretion are shown in **Figure 7**, IF = 0.937, indicating that the direct and indirect comparisons are in good agreement.

#### Cluster Analysis

**Figure 8:**
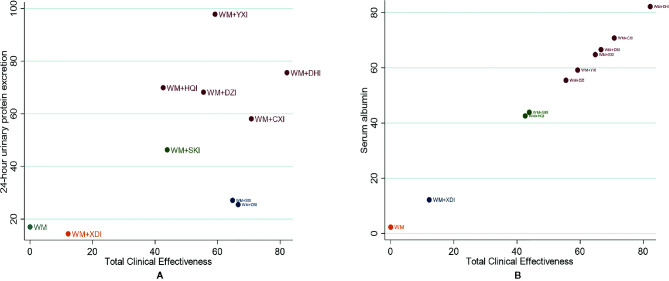
Cluster analysis plot for three outcomes. **(A)** Cluster analysis plot of total clinical effectiveness and 24h-UTP; **(B)** cluster analysis plot of Total clinical effectiveness and ALB. Interventions with the same color belonged to the same cluster, and interventions located in the upper right corner indicate optimal therapy for two different outcomes; WM, Western medicine; XDI, Xiangdan injection; HQI, Huangqi injection; SKI, Shenkang injection; DSI, Danshen injection; YXI, Yinxingdamo injection; DZI, Dengzhanhuasu injection; DHI, Danhong injection; SXI, Shuxuetong injection; CXI, Chuanxiongqin injection.

The cluster analysis method allowed for a comprehensive comparison of the effects of different interventions on TCE, 24-UTP, and ALB. The results showed (**Figure 8**) that WM+YXI and WM+DHI was the best intervention in terms of total clinical effectiveness and reducing 24-UTP, TCE and increasing ALB respectively.

#### Publication Bias

**Figure 9:**
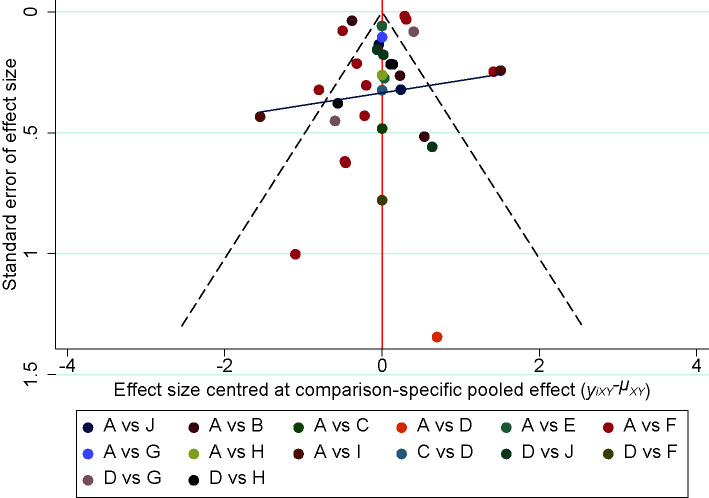
Funnel plot of the 24h-UTP. A, Western medicine; B, Chuanxiongqin injection; C, Danhong injection; D, Danshen injection; E, Dengzhanhuasu injection; F, Huangqi injection; G, Shenkang injection; H, Shuxuetong injection; I, Xiangdan injection; J, Yinxingdamo injection.

The comparison- adjusted funnel plots for each outcome measure were plotted and the scatter was found to be symmetrical along the null line to the left and right, so it was assumed that there was no small sample effect, of which 24h-UTP is shown in **Figure 9**.

### Security Evaluation

Of the 41 RCTs included, 30 RCTs reported safety indicators, of which adverse reactions were reported in 5 RCTs (Lin and Lai, 1999; Liu and Zhu, 2008; Tursun and Liang, 2010; Zhang, 2011; Fu and Zhang, 2014). Most of the adverse reactions in the combined CHIs treatment group were acne, hirsutism and other Cushing’s syndrome, leukopenia, infection, but also fever and facial flushing, mild hepatic impairment, and chest tightness. These symptoms can all recover spontaneously with symptomatic management or slowing down. Adverse effects in the control group treated with Western medicine alone also include signs or symptoms of Cushing’s syndrome, infection, and leukopenia, but the incidence is lower than in the treatment group. Other adverse reactions in the control group included nausea, abdominal discomfort, fever, etc., which were relieved by symptomatic treatment or slowing down the drip speed.

## Discussion

PNS usually has edema and proteinuria as its main clinical manifestations, which are related to various diseases in TCM. Generally speaking, those with edema as the main symptom can be categorized as “edema disease”. Also those with proteinuria as the main symptom can be categorized as “urine turbidity”. The main pathogenesis of nephrotic syndrome is spleen and kidney deficiency, stasis and stagnation of water, and the treatment is to “jianpigushen,xingshuihuayu”(to strengthen the spleen and consolidate the kidney, and to move water to eliminate stasis). The nine kinds of CHIs are extracted from single or multi-flavored herbs such as Astragalus, Leech, Salvia miltiorrhiza and other herbs which are beneficial to qi and blood, by modern scientific and technological means. They have been reliably and widely used for PNS in China. The results of pre-conventional meta-analysis shown that, relative to conventional western medical intervention protocols, the use of combined chinese and western medicine to treat PNS can effectively reduce clinical symptoms, promote recovery of relevant indicators (Li et al., 2018; Mo et al., 2018; Qu et al., 2019). However, the similarity of their efficacy and treatment and the unclear differences in efficacy lead to confusion in the choice of drugs. In this study, a network meta-analysis was used to achieve indirect comparisons of the effects of different types of dressings based on common controls. Results from both direct and indirect comparative evidence are combined; and the various types of herbal injections are quantitatively ranked in order to the optimal solution was obtained to provide a basis for selecting the appropriate CHIs for the treatment of PNS.

The results of network meta-analysis showed that different CHIs had different advantages in the adjuvant treatment of PNS. Among them, the combination of DHI and western medicine was most effective in increasing total clinical effectiveness and serum albumin. In terms of 24-h urinary protein excretion, WM+ YXI had the highest probability of being the best option, followed by WM+DHI and WM+HQI; in lowering cholesterol, the most effective combination of DZI with western medicine; WM+SYI was the most effective measure in lowering serum triglycerides; CXI combined with WM was the most effective in lowering serum triglycerides. The most significant efficacy is in the aspect of reducing fibrinogen. DHI contains two herbs, Salvia miltiorrhiza and Safflower (3:1 ratio) (Yale et al., 2015; Ma, 2020); YXI is a complex preparation of Ginkgo biloba extract (Zhou and Hou, 2013); DZI is a herbal preparation of Lanternflower with high clinical dosage (Ouyang et al., 2011); SXI is a compound injection made from two animal-based herbal extracts, leeches and Dilong (Yalei et al., 2015); CXI is an amide alkaloid extracted from Ligusticum Chuanxiong and refined by modern science and technology (Li et al., 2009). As can be seen in the above outcome indicators, the top-ranked CHI for treating PNS are all herbal medicines which can promote blood circulation and resolve blood stasis. PNS is often associated with varying degrees of local hemodynamic effects and even microcirculatory disturbances, with hypercoagulability of the blood and vascular disease (Oflaz et al., 2008; Gungor, 2013). The pharmacological effects and mechanism of traditional Chinese medicines with the effect of activating blood circulation and resolving stasis, such as Salvia miltiorrhiza, safflower, ginkgo biloba, leech, Chuanxiong rhizoma, etc., have been studied. Blood-vitalizing herbal medicine can improve blood rheology, hemodynamics, microcirculation, vascular regeneration, and antithrombosis through anti-improving hemodynamics antiplatelet effect, etc., effectively prevent the further development of PNS (Shu et al., 2006; Li and Wang, 2010; Qiu et al., 2018; MEIm et al., 2019). The results of this study indicated that WM alone was not as effective as WM+CHIs in the multiple group comparisons of six outcomes, such as total clinical effectiveness, 24-h urinary protein excretion, serum albumin, and lipids. And the SUCRA ranking of WM treatment alone was lower, so the results also reflect the benefit of combination of chinese and western medical therapy was superior to conventional western medical treatment alone to patients with PNS. Of the 41 studies included, only 5 specifically mentioned adverse reactions, 14 in total, most of which were either mild allergic symptoms or the result of an allergic reaction. It can be seen that the probability of adverse reactions to CHIs is low and has a good safety profile. Some TCM can also play a synergistic role in the treatment of PNS, reducing the effects of immunosuppressive drugs or glucocorticosteroids on the body (Li, 2017; Yuan et al., 2020).

## Innovations and Limitations of the Study

For the first time, this study used a network meta-analysis to compare the differences of XDI, HQI, SKI, DSI, YXI, DZI, DHI, SXI, and CXI in the clinical efficacy. And the nine CHIs were ranked in order of their superiority and inferiority. It provides high-level evidence to support the selection of herbal injections for clinical use. However, a quality assessment of the literatures included in this study showed that the methodological quality of the include studies were low. Only one articles reported whether or not blinding assignment, and all of the included documents did not indicate assignment concealment. All the included literature was in Chinese, and the lack of pre-study trial protocols from other countries were not disclosed in advance. The duration of treatment varied among studies and for ease of analysis, this study categorized diuretic and anticoagulant, anticoagulation, calcium supplementation, and lipid lowering as routine symptomatic treatment, to some extent, also led to clinical heterogeneity.

## Conclusion

In summary, the CHIs combined with WM therapy can bring greater benefits to PNS patients. In addition, this study has some limitations, therefore, the conclusions of this study need to be maintained with caution and more double-blinded multicenter, large-sample, high-quality randomized controlled trial are needed in the future.

## Author Contributions

HXY, MH, and WL performed the network meta-analysis. HXY and KY assessed the quality of the network meta-analysis. MH and LW analyzed study data. HXY and PL wrote the paper. MP made a significant contribution to the revision of the manuscript. HXY and MH contributed equally to this article. All authors contributed to the article and approved the submitted version.

## Funding

The National Natural Science Foundation of China (grant 81873263).

## Conflict of Interest

The authors declare that the research was conducted in the absence of any commercial or financial relationships that could be construed as a potential conflict of interest.
