# Supplementary material for: Efficacy of Chinese Herbal Injections for the Treatment of Primary Nephrotic Syndrome: A Bayesian Network Meta-Analysis of Randomized Controlled Trials
Source: Front Pharmacol. 2020 Oct 16;11:579241. doi: 10.3389/fphar.2020.579241 (PMC7596388; doi:10.3389/fphar.2020.579241)
Supplement: Supplementary file 1 [file Table_1.docx]

| **Section/topic** | **#** | **Checklist item** | **Reported on page #** |
| --- | --- | --- | --- |
| **TITLE** | | |  |
| Title | 1 | Identify the report as a systematic review, meta-analysis, or both. | 1 |
| **ABSTRACT** | | |  |
| Structured summary | 2 | Provide a structured summary including, as applicable: background; objectives; data sources; study eligibility criteria, participants, and interventions; study appraisal and synthesis methods; results; limitations; conclusions and implications of key findings; systematic review registration number. | 1-2 |
| **INTRODUCTION** | | |  |
| Rationale | 3 | Describe the rationale for the review in the context of what is already known. | 2-3 |
| Objectives | 4 | Provide an explicit statement of questions being addressed with reference to participants, interventions, comparisons, outcomes, and study design (PICOS). | 3 |
| **METHODS** | | |  |
| Protocol and registration | 5 | Indicate if a review protocol exists, if and where it can be accessed (e.g., Web address), and, if available, provide registration information including registration number. | / |
| Eligibility criteria | 6 | Specify study characteristics (e.g., PICOS, length of follow-up) and report characteristics (e.g., years considered, language, publication status) used as criteria for eligibility, giving rationale. | 4 |
| Information sources | 7 | Describe all information sources (e.g., databases with dates of coverage, contact with study authors to identify additional studies) in the search and date last searched. | 4 |
| Search | 8 | Present full electronic search strategy for at least one database, including any limits used, such that it could be repeated. | 4 |
| Study selection | 9 | State the process for selecting studies (i.e., screening, eligibility, included in systematic review, and, if applicable, included in the meta-analysis). | 4 |
| Data collection process | 10 | Describe method of data extraction from reports (e.g., piloted forms, independently, in duplicate) and any processes for obtaining and confirming data from investigators. | 5 |
| Data items | 11 | List and define all variables for which data were sought (e.g., PICOS, funding sources) and any assumptions and simplifications made. | 5 |
| Risk of bias in individual studies | 12 | Describe methods used for assessing risk of bias of individual studies (including specification of whether this was done at the study or outcome level), and how this information is to be used in any data synthesis. | 5 |
| Summary measures | 13 | State the principal summary measures (e.g., risk ratio, difference in means). | 5 |

| **Section/topic** | **#** | **Checklist item** | **Reported on page #** |
| --- | --- | --- | --- |
| Synthesis of results | 14 | Describe the methods of handling data and combining results of studies, if done, including measures of consistency (e.g., I^2^) for each meta-analysis. | 5 |
| Risk of bias across studies | 15 | Specify any assessment of risk of bias that may affect the cumulative evidence (e.g., publication bias, selective reporting within studies). | 5 |
| Additional analyses | 16 | Describe methods of additional analyses (e.g., sensitivity or subgroup analyses, meta-regression), if done, indicating which were pre-specified. | 5 |
| **RESULTS** | | |  |
| Study selection | 17 | Give numbers of studies screened, assessed for eligibility, and included in the review, with reasons for exclusions at each stage, ideally with a flow diagram. | 5-6 |
| Study characteristics | 18 | For each study, present characteristics for which data were extracted (e.g., study size, PICOS, follow-up period) and provide the citations. | 6 |
| Risk of bias within studies | 19 | Present data on risk of bias of each study and, if available, any outcome level assessment (see item 12). | 6 |
| Results of individual studies | 20 | For all outcomes considered (benefits or harms), present, for each study: (a) simple summary data for each intervention group (b) effect estimates and confidence intervals, ideally with a forest plot. | 6-9 |
| Synthesis of results | 21 | Present results of each meta-analysis done, including confidence intervals and measures of consistency. | 9-10 |
| Risk of bias across studies | 22 | Present results of any assessment of risk of bias across studies (see Item 15). | 10 |
| Additional analysis | 23 | Give results of additional analyses, if done (e.g., sensitivity or subgroup analyses, meta-regression [see Item 16]). | 10 |
| **DISCUSSION** | | |  |
| Summary of evidence | 24 | Summarize the main findings including the strength of evidence for each main outcome; consider their relevance to key groups (e.g., healthcare providers, users, and policy makers). | 10-12 |
| Limitations | 25 | Discuss limitations at study and outcome level (e.g., risk of bias), and at review-level (e.g., incomplete retrieval of identified research, reporting bias). | 12-13 |
| Conclusions | 26 | Provide a general interpretation of the results in the context of other evidence, and implications for future research. | 13 |
| **FUNDING** | | |  |
| Funding | 27 | Describe sources of funding for the systematic review and other support (e.g., supply of data); role of funders for the systematic review. | 13 |

*From:*  Moher D, Liberati A, Tetzlaff J, Altman DG, The PRISMA Group (2009). Preferred Reporting Items for Systematic Reviews and Meta-Analyses: The PRISMA Statement. PLoS Med 6(7): e1000097. doi:10.1371/journal.pmed1000097

For more information, visit: **www.prisma-statement.**

| More details about the product information of 9 CHIs | | | | | | | | |
| --- | --- | --- | --- | --- | --- | --- | --- | --- |
| Injection name | Source | Species / Raw materials | Botanical plant names | Therapeutic claims in TCM | Indications | Adverse drug reactions | Quality control reported? (Y/N) | Chemical analysis reported? (Y/N) |
| Xiangdan injection | Tonghua Maoxiang Pharmaceutical Co., Ltd. | salvia miltiorrhiza；rosewood | Salvia miltiorrhiza Bge.； Dalbergia odorifera T.Chen | / | Dilates blood vessels and enhances coronary blood flow. Used for angina pectoris, can also be used for myocardial infarction. | 1. Systemic damage: anaphylactic reaction, anaphylactic shock, cyanosis, fever, chills, syncope, etc.  2. Respiratory system damage: dyspnea, chest tightness, cough, wheezing, laryngeal edema and so on.  Cardiovascular system damage: palpitations.  Damage to the central and peripheral nervous system: dizziness, headache.  Damage to the skin and its accessories: rash, itching.  6. Damage to the gastrointestinal system: nausea, vomiting. | Y - YBZ09182004 issued by China Food and Drug Administration | N |
| Huangqi injection | Chitai Qingchunbao Pharmaceutical Co., Ltd. | astragalus. | Astragalus memeranaceus (Fisch.) Bge. var. mongholicus (Bge.) Hsiao | Benefiting the vital energy and nourishing the body, strengthening the heart and clearing the vena cava, strengthening the spleen and inducing dampness. | Viral myocarditis and cardiac insufficiency due to heart-Qi deficiency and blood stasis.AND Hepatitis due to spleen deficiency and dampness. | 1. Allergic reactions: drug fever, drug rash, redness and swelling at the injection site are common; rarely, acute allergic reactions and anaphylaxis are severe. Adverse Reactions.  2. Respiratory system: common laryngeal edema, dyspnea, asthma, chest tightness.  3. Circulatory system: Occasionally hypotension with delayed phlebitis; rarely rapid atrial fibrillation.  4. Digestive system: occasionally liver function impairment, vomiting, diarrhea.  Other: Occasionally severe headache, renal impairment, rare hemolytic anemia, pyrogenic reaction to intravenous drops of the product has been reported. | Y –  Z33020178 issued by China Food and Drug Administration | N |
| Shenkang  injection | Xi'an Shiji Shengkang Pharmaceutical Co., Ltd. | rhubarb； salvia miltiorrhiza; Carthami Flos; astragalus. | Rheum palmatum, L.; Salvia miltiorrhiza Bge.； Carthamus tinctorius L.; Astragalus memeranaceus (Fisch.) Bge. | subdues rebelliousness and drains turbidity, invigorates Qi and blood, and expels dampness from the internal organs. | suitable for chronic renal failure caused by dampness and blood stasis. | Local irritation such as redness, pain, pruritus, rash and thirst are occasionally seen during static drops. | Y - YBZ08522004 issued by China Food and Drug Administration | N |
| Danshen injection | Chitai Qingchunbao Pharmaceutical Co., Ltd. | Salvia miltiorrhiza | Salvia miltiorrhiza Bge. | Promoting blood circulation and relieving blood stasis, invigorating the veins and nourishing the heart. | Coronary heart disease, chest tightness, angina | Occasional allergic reactions are seen. | Y -  Z33020177 issued by China Food and Drug Administration | N |
| Yinxingdamo injection | Guizhou Yibai Pharmaceutical Co., Ltd. | Ginkgo biloba Extract | Ginkgo biloba L. | / | Prevention and treatment of coronary heart disease, thromboembolic diseases | 1、Sometimes nausea, vomiting, dizziness, skin allergic reactions occur.  2.Rarely, angina worsens, but the symptoms disappear immediately when the drug is stopped.  3.Anaphylactic shock has been reported to occur. | Y -  H52020032 issued by China Food and Drug Administration | N |
| Dengzhanhuasu  injection | Yunnan Bio Valley Pharmaceutical Co.,Ltd | Erigeron breviscapus | Erigeron breviscapus (Vant.) Hand. -Mazz. | Promoting blood circulation and removing blood stasis, Tongmai analgesia | Stroke sequelae, coronary heart disease, angina | Occasionally itching, chest tightness, fatigue, rash, palpitations, etc. | Y -  Z20043108 issued by China Food and Drug Administration | N |
| Danhong injection | Heze Buchang Pharmaceutical Co., Ltd. | salvia miltiorrhiza; Carthami Flos; | Salvia miltiorrhiza Bge.； Carthamus tinctorius L.; | Invigorate the circulation of blood and remove blood stasis | chest paralysis and strokes caused by Blood stasis and obstruction. | occasionally has allergic reactions, which may include rash, itching, headache, dizziness, palpitations, chills, fever, facial flushing, nausea, vomiting, and Diarrhea, chest tightness, dyspnea, laryngeal edema, convulsions, etc., all return to normal after discontinuation of the drug. Rarely anaphylactic shock. | Y -  Z20026866 issued by China Food and Drug Administration | N |
| Shuxuetong  injection | Mudanjiang Youbo Pharmaceutical Co.,Ltd | Leeches, Dilophosaurus | / | Promoting blood circulation and removing blood stasis | the acute phase of stroke caused by blood stasis and obstruction of the meridians | Allergic reactions: generalized skin flushing, rash, pruritus, hives, laryngeal edema, dyspnea, breathlessness, palpitations, cyanosis, decreased blood pressure, anaphylactic shock, etc.  Systemic damage: chills, fever, hyperthermia, chills, fatigue, etc.  Respiratory system: chest tightness, dyspnea, shortness of breath, cough, breath-holding, etc.  Cardiovascular system: palpitations, etc.  Digestive system: nausea, vomiting, abdominal pain, diarrhea, etc.  Nervous system: dizziness, headache, convulsions, etc.  Skin and accessories: rash, hives, macules, erythema, pruritus, sweating, etc.  Other: purpura, hematuria, gastrointestinal bleeding, conjunctival hemorrhage, subcutaneous hemorrhage, abnormal prothrombin time, etc.; there are case reports of thrombocytopenia and ocular hemorrhage. | Y -  Z20010100 issued by China Food and Drug Administration | N |
| Chuanxiongqin  injection | Shijiazhuang Yiling Pharmaceutical Co., Ltd. | Ligusticum wallichii | Ligusticum chuam. iong Hort. | / | For occlusive cerebrovascular diseases such as cerebral insufficiency, cerebral thrombosis, cerebral embolism. | This product has strong acidity and strong irritant point injection. | Y -  H20063094 issued by China Food and Drug Administration | N |
