# Supplementary material for: Efficacy of Chinese Herbal Injections for the Treatment of Primary Nephrotic Syndrome: A Bayesian Network Meta-Analysis of Randomized Controlled Trials
Source: Front Pharmacol. 2020 Oct 16;11:579241. doi: 10.3389/fphar.2020.579241 (PMC7596388; doi:10.3389/fphar.2020.579241)
Supplement: Supplementary file 2 [file Table_2.docx]

| Specific intervention methods of the study | | |
| --- | --- | --- |
| Study ID | Interventions | |
|  | Treatment group | Control group |
| **Ye,2006** | On the basis of the control group, add XDI intravenously | Conventional treatment: prednisone 1mg/(kg/d), take it in the morning, gradually reduce the dose in 8-12 weeks, and give symptomatic treatment, such as anti-infection and blood pressure reduction. |
| **Li,2017** | On the basis of the control group, add XDI intravenously | Conventional treatment: prednisone 1mg/(kg/d), take it in the morning, gradually reduce the dose in 8-12 weeks, and give symptomatic treatment, such as anti-infection and blood pressure reduction. |
| **Sun,2015** | On the basis of the control group, add HQI intravenously | Conventional treatment: prednisone 1mg/(kg/d), take it in the morning, and give symptomatic treatment, such as anti-infection and blood pressure reduction. |
| **Chen,2015** | On the basis of the control group, add HQI intravenously | Conventional treatment: prednisone 1mg/(kg/d), take it in the morning, and give symptomatic treatment, such as anti-infection and blood pressure reduction. |
| **Liu,2014** | On the basis of the control group, add HQI intravenously | Conventional treatment: prednisone 1mg/(kg/d), take it in the morning, gradually reduce the dose in 8-12 weeks, and give symptomatic treatment, such as anti-infection and blood pressure reduction. |
| **Gong,2014** | On the basis of the control group, add HQI intravenously | Conventional treatment: prednisone 1mg/(kg/d), take it in the morning, gradually reduce the dose in 8-12 weeks, and give symptomatic treatment, such as anti-infection and blood pressure reduction. |
| **Fu,2014** | Oral glucocorticoids in all patients: prednisone acetate (lm gd/kg) maximum dose not exceeding 60 mg/d) and given diuresis, lipid-lowering, blood pressure (taking ACEI drugs) and other basic treatments, on this basis, SKI intravenous infusion was added. | Oral glucocorticoids in all patients: prednisone acetate (l mgd/kg) maximum dose not exceeding 60 mg/d) and given diuresis, lipid-lowering, blood pressure (taking ACEI drugs) and other basic treatments, on this basis, DSI intravenous infusion was added. |
| **Li,2013** | Prednisone [1mg·(kg·d)-1] standard treatment according to the patient’s condition, with diuretics and other conventional treatments if necessary. on this basis, YXI intravenous infusion was added. | Prednisone [1mg·(kg·d)^-1^] standard treatment according to the patient’s condition, with diuretics and other conventional treatments if necessary. on this basis, DSI intravenous infusion was added. |
| **Cai,2013** | On the basis of the control group, add DZI intravenously. | After admission, patients were given routine nephrotic syndrome treatment and adequate prednisolone at 1 m/kg per day. |
| **Wang,2012** | On the basis of the control group, add YXI intravenously. | Conventional treatment: prednisone 1mg/(kg/d), and give symptomatic treatment, such as anti-infection and blood pressure reduction. |
| **Lei,2012** | On the basis of the control group, add HQI intravenously. | Conventional treatment: prednisone 1mg/(kg/d), take it in the morning, gradually reduce the dose in 8-12 weeks, and give symptomatic treatment, such as anti-infection and blood pressure reduction. |
| **Zhang,2011** | Conventional treatment: prednisone 1mg/(kg/d), take it in the morning, gradually reduce the dose in 8-12 weeks, and give symptomatic treatment, such as anti-infection and blood pressure reduction. on this basis, YXI intravenous infusion was added. | Conventional treatment: prednisone 1mg/(kg/d), take it in the morning, gradually reduce the dose in 8-12 weeks, and give symptomatic treatment, such as anti-infection and blood pressure reduction. on this basis, DSI intravenous infusion was added. |
| **Zhang,2011** | On the basis of the control group, add DZI intravenously. | Conventional treatment: prednisone 1mg/(kg/d), take it in the morning, gradually reduce the dose in 8-12 weeks, and give symptomatic treatment, such as anti-infection and blood pressure reduction. |
| **Yang,2011** | On the basis of the control group, add DHI intravenously. | Conventional treatment: prednisone 1mg/(kg/d), take it in the morning, gradually reduce the dose in 8-12 weeks, and give symptomatic treatment, such as anti-infection and blood pressure reduction. |
| **Xie,2011** | Conventional treatment: prednisone 1mg/(kg/d), take it in the morning, gradually reduce the dose in 8-12 weeks, and give symptomatic treatment, such as anti-infection and blood pressure reduction. on this basis, DKI intravenous infusion was added. | Conventional treatment: prednisone 1mg/(kg/d), take it in the morning, gradually reduce the dose in 8-12 weeks, and give symptomatic treatment, such as anti-infection and blood pressure reduction. on this basis, DSI intravenous infusion was added. |
| **Li,2011** | On the basis of the control group, add HQI intravenously. | Patients were treated with lisinopril 10mg/d (ACEI drugs) + prednisolone 1mg/kg/d (glucocorticoids drugs) and taken with chinton. |
| **Yang,2010** | On the basis of the control group, add DHI intravenously. | Patients were treated with glucocorticoid in addition to cyclophosphamide, 2 ~ 3 mg/kg per day, and the total amount of cyclophosphamide should not exceed 8g. |
| **Song,2010** | Conventional treatment: prednisone 1mg/(kg/d), take it in the morning, gradually reduce the dose in 8-12 weeks, and give symptomatic treatment, such as anti-infection and blood pressure reduction. on this basis, SKI intravenous infusion was added. | Conventional treatment: prednisone 1mg/(kg/d), take it in the morning, gradually reduce the dose in 8-12 weeks, and give symptomatic treatment, such as anti-infection and blood pressure reduction. on this basis, DSI intravenous infusion was added. |
| **Zhou,2009** | On the basis of the control group, add HQI intravenously. | Conventional treatment: prednisone 1mg/(kg/d), take it in the morning, gradually reduce the dose in 8-12 weeks, and give symptomatic treatment, such as anti-infection and blood pressure reduction. |
| **Yuan,2009** | On the basis of the control group, add SXI intravenously. | Patients were given a high quality low-salt and low-protein diet, those with severe edema were given diuretics, and antibiotics were routinely used in patients with coinfection. |
| **Yuan,2009** | On the basis of the control group, add DSI intravenously. | Conventional treatment: prednisone 1mg/(kg/d), take it in the morning, gradually reduce the dose in 8-12 weeks, and give symptomatic treatment, such as anti-infection and blood pressure reduction. |
| **Dai,2009** | On the basis of the control group, add CXI intravenously. | Conventional treatment: prednisone 1mg/(kg/d), take it in the morning, gradually reduce the dose in 8-12 weeks, and give symptomatic treatment, such as anti-infection and blood pressure reduction. |
| **Xv,2008** | On the basis of the control group, add HQI intravenously. | Conventional treatment: prednisone 1mg/(kg/d), take it in the morning, gradually reduce the dose in 8-12 weeks, and give symptomatic treatment, such as anti-infection and blood pressure reduction. |
| **Liu,2008** | On the basis of the control group, add HQI intravenously. | The standard dose of Prednisone (1mg· kg^-1^ ·D^-1^) was taken on a morning basis for 45 consecutive days. Cyclophosphamide was injected intravenously and the total amount was 8 ~ l0g. Low molecular weight heparin calcium 4100U subcutaneous injection, once a day, 45 days for a course of treatment. |
| **Turkson,**  **2010** | On the basis of the control group, add YXI intravenously. | Conventional treatment: prednisone 1mg/(kg/d), take it in the morning, gradually reduce the dose in 8-12 weeks, and give symptomatic treatment, such as anti-infection and blood pressure reduction. |
| **Li,2007** | Conventional treatment: prednisone 1mg/(kg/d), take it in the morning, gradually reduce the dose in 8-12 weeks, and give symptomatic treatment, such as anti-infection and blood pressure reduction. on this basis, SKI intravenous infusion was added. | Conventional treatment: prednisone 1mg/(kg/d), take it in the morning, gradually reduce the dose in 8-12 weeks, and give symptomatic treatment, such as anti-infection and blood pressure reduction. on this basis, DSI intravenous infusion was added. |
| **Deng,2007** | Conventional treatment: prednisone 1mg/(kg/d), take it in the morning, gradually reduce the dose in 8-12 weeks, and give symptomatic treatment, such as anti-infection and blood pressure reduction. on this basis, YXI intravenous infusion was added. | Conventional treatment: prednisone 1mg/(kg/d), take it in the morning, gradually reduce the dose in 8-12 weeks, and give symptomatic treatment, such as anti-infection and blood pressure reduction. on this basis, DSI intravenous infusion was added. |
| **Chu,2011** | On the basis of the control group, add SXI intravenously. | Conventional treatment: prednisone 1mg/(kg/d), take it in the morning, gradually reduce the dose in 8-12 weeks, and give symptomatic treatment, such as anti-infection and blood pressure reduction. |
| **Ma,2002** | On the basis of the control group, add DSI intravenously. | Patients were treated with a standard course of glucocorticoid with prednisone 1mg·(kg·d)-1 dose of chinton. |
| **Zhang,2016** | On the basis of the control group, add HQI intravenously. | Conventional treatment: prednisone 1mg/(kg/d), take it in the morning, gradually reduce the dose in 8-12 weeks, and give symptomatic treatment, such as anti-infection and blood pressure reduction. |
| **Liu,2008** | Conventional treatment: prednisone 1mg/(kg/d), take it in the morning, gradually reduce the dose in 8-12 weeks, and give symptomatic treatment, such as anti-infection and blood pressure reduction. on this basis, HQI intravenous infusion was added. | Conventional treatment: prednisone 1mg/(kg/d), take it in the morning, gradually reduce the dose in 8-12 weeks, and give symptomatic treatment, such as anti-infection and blood pressure reduction. on this basis, DSI intravenous infusion was added. |
| **Niu,2004** | Conventional treatment: prednisone 1mg/(kg/d), take it in the morning, gradually reduce the dose in 8-12 weeks, and give symptomatic treatment, such as anti-infection and blood pressure reduction. on this basis, SXI intravenous infusion was added. | Conventional treatment: prednisone 1mg/(kg/d), take it in the morning, gradually reduce the dose in 8-12 weeks, and give symptomatic treatment, such as anti-infection and blood pressure reduction. on this basis, DSI intravenous infusion was added. |
| **Bai,2004** | On the basis of the control group, add HQI intravenously. | Conventional treatment: prednisone 1mg/(kg/d), take it in the morning, gradually reduce the dose in 8-12 weeks, and give symptomatic treatment, such as anti-infection and blood pressure reduction. |
| **Zhang,2003** | On the basis of the control group, add DSI intravenously. | Patients were given low-molecular-weight dextran, 500ml, once a day; oral hydrochlorothiazide 25mg, twice a day; severe edema add triamteridine, 50mg, twice a day; and oral prednisone 2mg/ (kg ·d). |
| **Yang,2002** | On the basis of the control group, add HQI intravenously. | Conventional treatment: prednisone 1mg/(kg/d), take it in the morning, gradually reduce the dose in 8-12 weeks, and give symptomatic treatment, such as anti-infection and blood pressure reduction. |
| **Shen,2002** | On the basis of the control group, add HQI intravenously. | Conventional treatment: prednisone 1mg/(kg/d), take it in the morning, gradually reduce the dose in 8-12 weeks, and give symptomatic treatment, such as anti-infection and blood pressure reduction. |
| **Lin,2005** | On the basis of the control group, add HQI intravenously. | Conventional treatment: prednisone 1mg/(kg/d), take it in the morning, gradually reduce the dose in 8-12 weeks, and give symptomatic treatment, such as anti-infection and blood pressure reduction. |
| **Long,2018** | On the basis of the control group, add SKI intravenously. | Prednisone acetate tablets and cyclophosphamide tablets |
| **Zhuang,**  **1998** | On the basis of the control group, add CXI intravenously. | Conventional treatment: prednisone 1mg/(kg/d), take it in the morning, gradually reduce the dose in 8-12 weeks, and give symptomatic treatment, such as anti-infection and blood pressure reduction. |
| **Li,2016** | On the basis of the control group, add CXI intravenously. | Conventional treatment: prednisone 1mg/(kg/d), take it in the morning, gradually reduce the dose in 8-12 weeks, and give symptomatic treatment, such as anti-infection and blood pressure reduction. And mycophenolate mofetil dispersible tablet was taken orally, 1.0 g/time, 2 times/d. |
| **Feng,2010** | Conventional treatment: prednisone 1mg/(kg/d), take it in the morning, gradually reduce the dose in 8-12 weeks, and give symptomatic treatment, such as anti-infection and blood pressure reduction. on this basis, DSI intravenous infusion was added. | Conventional treatment: prednisone 1mg/(kg/d), take it in the morning, gradually reduce the dose in 8-12 weeks, and give symptomatic treatment, such as anti-infection and blood pressure reduction. on this basis, DHI intravenous infusion was added. |
